# An Optimized Framework for Energy-Resource Allocation in a Cloud Environment based on the Whale Optimization Algorithm

**DOI:** 10.3390/s21051583

**Published:** 2021-02-24

**Authors:** Shanky Goyal, Shashi Bhushan, Yogesh Kumar, Abu ul Hassan S. Rana, Muhammad Raheel Bhutta, Muhammad Fazal Ijaz, Youngdoo Son

**Affiliations:** 1 Research Scholar, CSE Department, IKGPTU, Jalandhar 144603, India; shanky.it@cgc.edu.in; 2 IT Department, Chandigarh Group of Colleges, Landran, Punjab 140307, India; hod.it@cgc.edu.in; 3 CSE Department, Chandigarh Group of Colleges, Landran, Punjab 140307, India; Yogesh.rise@cgc.edu.in; 4 Department of Intelligent Mechatronics Engineering, Sejong University, Seoul 05006, Korea; rana@sejong.ac.kr (A.u.H.S.R.); fazal@sejong.ac.kr (M.F.I.); 5 Department of Computer Science and Engineering, Sejong University, Seoul 05006, Korea; 6 Department of Industrial and Systems Engineering, Dongguk University-Seoul, Seoul 04620, Korea

**Keywords:** load balancing, energy efficiency, resource scheduling, power consumption, cloud computing, whale optimization

## Abstract

Cloud computing offers the services to access, manipulate and configure data online over the web. The cloud term refers to an internet network which is remotely available and accessible at anytime from anywhere. Cloud computing is undoubtedly an innovation as the investment in the real and physical infrastructure is much greater than the cloud technology investment. The present work addresses the issue of power consumption done by cloud infrastructure. As there is a need for algorithms and techniques that can reduce energy consumption and schedule resource for the effectiveness of servers. Load balancing is also a significant part of cloud technology that enables the balanced distribution of load among multiple servers to fulfill users’ growing demand. The present work used various optimization algorithms such as particle swarm optimization (PSO), cat swarm optimization (CSO), BAT, cuckoo search algorithm (CSA) optimization algorithm and the whale optimization algorithm (WOA) for balancing the load, energy efficiency, and better resource scheduling to make an efficient cloud environment. In the case of seven servers and eight server’s settings, the results revealed that whale optimization algorithm outperformed other algorithms in terms of response time, energy consumption, execution time and throughput.

## 1. Introduction

Cloud computing represents a fusion of two major trends that are IT efficiency and business agility. The cloud has the provision of storing data without any limitation as well as hiding a tremendous volume of data from other users. The users can access the required files, documents and applications on demand. Users only pay for the services provided by the cloud instead of buying the own expensive infrastructure. Cloud computing has various features such as on-demand resource allocation, quality of service, elasticity, etc. which makes this very engaging both in the academic as well as the business domain. The continual demand for services provided by the cloud has led to the need to manage the load of machines, energy produce by that machines and scheduling of resources. Load balancing is the technique of allocating different tasks over different resources in the data center to maintain balance [1]. The resources available can be a data center, a virtual machine, or, a physical machine [2,3].

The resource and service dispensation must be done in a systematic way so that every resource should experience the same loads at any instant of time and should improve the average utilization rate of resources [4]. If there is any kind of load imbalance, then the system performance will be drastically decreased. While maintaining the load, energy consumption should also be kept in mind. Green cloud computing is a term that covers utilization of resources efficiently as well as reduces energy consumed by these resources [5]. The energy consumption resources in the datacenter are both due to cooling as well as computational resources. While the computational resources cover around 60% of the total energy consumption the other 30% is covered by the cooling infrastructure [6]. 

**Figure 1:**
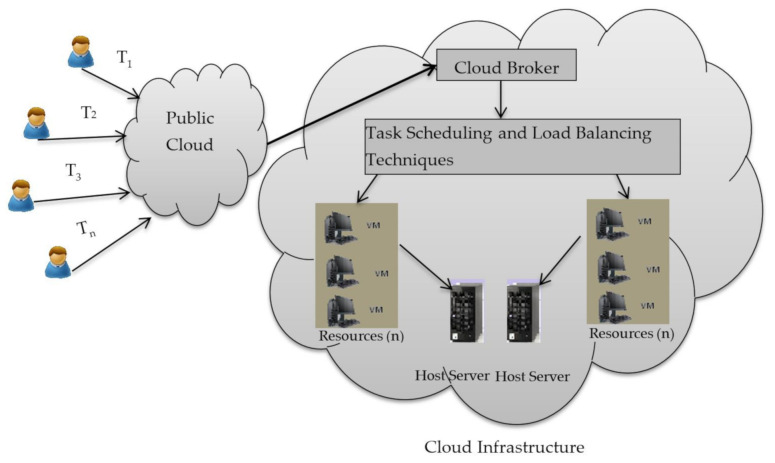
Cloud Infrastructure Bala [7].

The energy consumption problem can be divided into two parts: (a) First, one with server-side operations, and, (b) networking side communications. Optimizing resource allocation as well as reducing the operational cost is a key concept. This can be implemented in the platform as a service segment. Schedulers are used to schedule the resources and the load balancers are used to balance the resources and to predict the load as well as to reduce the energy. In case of cloud computing, the services or resources are either allocated or de allocated. The major benefit of incorporating into cloud is that it removes the pressure of upfront investment and hence lowers the cost of operation and maintenance. Also, Figure 1 below depicts a demonstration of cloud infrastructure. The scalability of cloud computing provides users with a level of flexibility and can be scaled up and down according to the need of users. Resources allocation is the task of allocating the resources while maintaining the proper balance in the environment. To maintain a proper balance, resource scheduling algorithms are applied to get an efficient performance.

Nowadays fog computing and edge computing which are extensions of cloud computing are also used, because IOT devices use cloud computing for storage of data. Fog computing helps to reduce the network burden of data centers and edge computing is used to minimize and manage the traffic [8,9]. A lot of work has already been done on cloud systems using various optimization algorithms but there is still a need for improvement due to the increase in cloud system usage on a daily basis. The number of users is increasing, so companies are using more and more servers to make their services better, but this additional usage is also increasing the load, energy consumption and resources needed in the cloud system.

Hence, the key motivation behind this research was to improve various aspects of the performance of cloud systems like load balancing, resource scheduling, energy consumption using a novel metaheuristic approach named whale optimization algorithm (WOA). Before implementing the WOA algorithm, we perform an experimental survey on various algorithms that are good for load balancing, resource scheduling and energy efficiency. We used particle swarm optimization (PSO) and cuckoo search algorithm (CSA) algorithm for balancing the load over the clod system and calculate the corresponding values. Then we test CSO and BAT algorithm, which was doing good task-resource allocation as per literature and get their result for resource scheduling. Finally, we propose and implement the whale optimization algorithm which gives the best result for task execution, response time and energy consumption for a cloud system.

In the first phase, we implement the two algorithms PSO and CSA. PSO is a useful algorithm to allocate loads to different machines that is based on the social behaviors of animals as they find their food. PSO is useful to find the machines which have less load and assign tasks to that particular machine but the algorithm also has some limitations in that it takes more execution time [7]. The CSA algorithm is based on the strategy of cuckoos to lay eggs in the best nest and this strategy of cuckoos helps find the best machine for task allocation so that loads can be balanced properly [10]. This algorithm is best suited for job scheduling but the algorithm has limitations like resource scheduling. Both these algorithms give best results for load balancing but are not good for resource scheduling. Then in a second phase, implementation of the cat swarm optimization (CSO) and BAT has been done. The CSO algorithm uses the concept of cats based in two modes—seeking and tracing—which aims to efficiently allocate the available resources to a number of tasks in a cloud environment with minimum cost [11]. This algorithm gives good task-resource allocation strategy results. The bat algorithm is based on the strategy used by bats to catch them pray [12]. This approach is implemented to allocate the resources to tasks in such way that resources can be successfully scheduled using less budget and time, but it does not give good results as far as energy consumption is concerned. Both BAT and CSO give better results as compared to PSO and CSA. 

Lastly, the whale optimization algorithm (WOA) algorithm that gives the best result as compared to all algorithms that were implemented in previous phases has been implemented [13]. WOA starts with a random solution by considering the current solution is the best solution and based on that position the population is updated. It is based on the strategy of exploitation and exploration. The WOA algorithm gives comparative results for load balancing, resource scheduling and energy efficiency of cloud systems. For this testing, Cloud Analyst has been used. 

This paper includes various sections that cover a brief introduction to cloud computing, the proposed algorithms and results. Section 1 covers the introduction to the cloud concept. Section 2 is a review of the load balancing, energy efficiency and resource scheduling literature. Section 3 includes the proposed algorithms, while Section 4 shows the simulation results of our research and compares the results of existing and the proposed algorithms. Finally, Section 5 gives the conclusions and future scope of the research.

## 2. Related Work

Here we present a detailed review of recently developed systems based on resource scheduling and energy optimization. Also, we provide a review of existing systems that use various techniques to perform load balancing in the cloud.

### 2.1. Recently Developed Load Balancing Mechanisms

The subsequent section lists and describes different load balancing mechanisms that have been recently developed. Bhargavi et al. [14] recommended a scheme for public clouds that employs a raven roosting optimization policy (RROP) to achieve load balancing. The RROP architecture consists of two functional modules, namely, task-subset formulator (TF), and Physical Machine formulator (PMF). Liu et al. [15] presented PMK-ELM based on the K-extreme learning machine algorithm and TS-non-dominated sorting genetic algorithm version II based on NSGA-II which are integrated into a heterogeneous cloud environment. The PMK-ELM facilitates the prediction of task execution time while TS-NSGA-II facilitates the selection of suitable reducers. Jain et al. [2] proposed a load balancing approach that is designed in multiple phases for the cloud environment. The system demonstrates a two-level scheduler (JIJS) which combines: (a) join shortest queue approach, and, (b) join idle queue approach. It involves three main steps: (a) a scheduler with the maximum idle queue length is selected from the table, the assignment of the job is done and, the necessary updates are performed, (b) if the queue of all schedulers is void, then a VM with the least queue length is selected to assign the job, and, (c) on task completion, VM updates the VM table, and, finally the scheduler make suitable updates in the scheduler table. Joshi et al. [16] presented a new technique to relocate the load from an overloaded virtual machine to a VM that is under loaded. This technique helps in avoiding the swift load allocation on the under loaded machines. It results in an efficient resource utilization. It lets a suitable continuation with service level agreement. Faustina et al. [17] proposed a self-governing agent-based mechanism for balancing the workload of virtual machines using an independent agent. The load balancing task is performed by an autonomous migration agent that efficiently balances the workload. The SGA_LB algorithm mainly consists of three agents: (a) in-house agent, (b) external agent, and, (c) migration agents. When a machine gets overloaded, the in-house agent finds another suitable VM that resides on another data center. Kumar et al. [18] presented a flexible approach for balancing the workload. It employs a multi-time-based approach. The MTBLB algorithm utilizes many techniques in a single algorithm namely, aws cloud watch, algorithm for crone jobs, and the round-robin algorithm. Initially, when the watch is set to 0, all the requests begin to operate. Further, crone jobs are used for processing followed by a round-robin algorithm that is applied so that no starvation occurs and the whole operation time is reduced.

### 2.2. Energy Optimization in the Cloud

Energy utilization of a data center also produces various environmental issues [19,20]. These issues can be solved by calculating the consumption of equipment at regular intervals in real time for immediate and future processing. This section presents a review on various energy reduction techniques and evaluation methods. Duan et al. [21] proposed a prediction scheme which may predict the future length of intervals when a CPU is idle; it selects the most successful sleep state to reduce power consumption at run-time. It uses statistical models that predict the interval lengths. Goyal et al. [22] suggested a hybrid efficient method to reduce the consumption of energy in the cloud as well as ensuring QoS by reducing the SLA breaches. The approach uses two policies to reduce energy consumption namely, hybrid VM selection policy and low utilization host policy. This technique reduces the consumption of energy and CO_2_ emissions that significantly decreases health-related problems. Jiang et al. [23] presented a model for optimization that aims at decreasing the consumption of energy where data centers are dispensed area wise. Also, an intelligent heuristic algorithm called IOEN is proposed to handle dynamic requests between the DCs as well as between the DCs and users. This is executed using the combination of two algorithms namely: a) niche genetic algorithm, and, b) random depth-first search. Jasuja et al. [24] proposed a hybrid approach for optimizing the energy that produces good results in migration and energy consumption terms. The proposed system uses a combination of algorithms, namely ACO and a gravitational search algorithm. ACO aids in solving NP-hard problems and is utilized for dynamic VM-consolidation while the gravitational search algorithm is a local search algorithm that employs the law of gravity in which all agents tend to move towards the agent with a high gravitational mass which is considered as the best agent. The results infer that the method significantly improves energy optimization in a cloud environment. Khan et al. [25] introduced an energy optimization method that exploits information entered by the user in dynamic VM consolidation. An algorithm named release time-based DVMC (RTDVMC) is proposed which minimizes energy consumption. This algorithm is applied mainly in two phases, where the first phase deals in limiting the violations of SLA while the second phase comprises relocating the VMs and merging them in lesser PMs that are active. Majeed et al. [26] has done a survey on various techniques that can help to reduce energy consumption in the cloud environment. Various applications over the cloud consume high amount of energy but using integrated techniques and this way energy consumption can be reduced.

### 2.3. Resource Scheduling in a Cloud Based Environment

**Figure 2:**
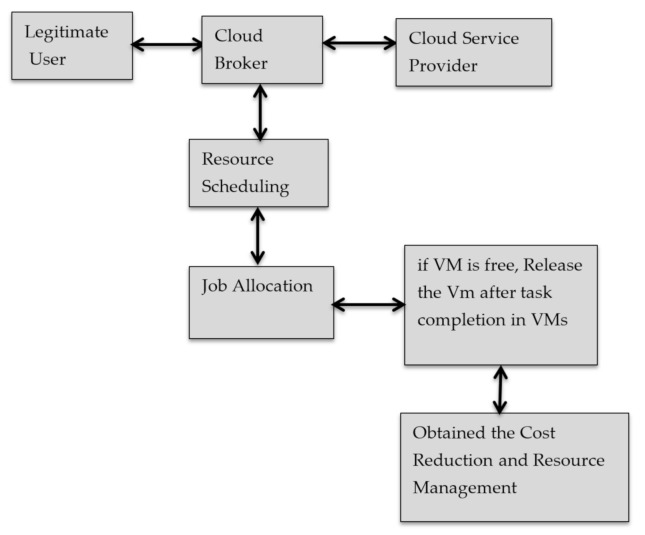
Workflow of Resource Scheduling Santhiya et al. [27].

To make work be efficient, proper scheduling of resources is a must. For this, there is need to apply resource scheduling algorithms which check that jobs are properly allocated only to those VMs which are free and also free up VMs after completion of tasks Santhiya et al. [27] as shown in Figure 2. Some of the existing works based on the scheduling of resources are reviewed in the section below.

Zhu et al. [28] recommended a 3D virtual resource scheduling technique in that is subdivided into following parts, namely, (a) virtual resource allocation, (b) virtual resource scheduling, and, (c) virtual resource optimization. The method reduces power as well as analyses how to keep cloud data centers balanced. Samadi et al. [29] proposed an algorithm to balance the workload known as DT-MG. The algorithm works in a certain fashion: (a) basic task scheduling is designed, (b) the under loaded as well as overloaded PMs are identified, (c) selection of the tasks that are to be migrated, and, (d) finally it chooses the best migration targets. Hassan et al. [30] presented an energy-efficient, scalable cloud resource allocation model for the processing of monitored body-censored data in real time. It works by employing two main models: (a) a LP model that applies linear programming which substantially optimizes the allocation of VM, and, (b) formulation to model virtual machine allocation that solves the issues of resource allocation by using different heuristics that are first—fit decreasing (FFD), best—fit decreasing (BFD), and worst-fit decreasing (WFD). Karpagam et al. [31] presented a heuristic and a novel shuffled frog leaping (SFLA) algorithm for workflow scheduling. The proposed technique is compared with SFLA without clustering and opportunistic load balancing (OLB). This algorithm keeps every computer busy while the SFLA method is inspired by the natural behavior of a group of frogs as they look for sources full of food. Raj et al. [32] recommended a two pass scheduling policy (TPSP) for resource scheduling using map reduce and Hadoop. This algorithm consists of two phases where the first pass focuses on building a scheduling queue list plus the same list is saved as a temporary list while the second pass emphasizes a temporary list. Pourghaffari et al. [33] also defined a resources allocation method based on cloud computing with a load balancing approach. The data center selection is executed using the fuzzy algorithm followed by the load balancing in the system performed using the DVFS algorithm, and the task scheduling that is done with the EDF-VD algorithm. Kumar et al. [34] presented a system that uses a cloud service provider which dispenses the load fairly amongst the existing VMs. The system begins the load balancing operation by calculating the load at VM and at the DC at a specific point in time and further it finds the capacity of each VM and data center. 

Table 1 provides the comparison of various techniques that are used in cloud environment for load balancing, resource scheduling and efficiently managing energy.

## 3. Proposed Work

### 3.1. Working Methodology 

**Figure 3:**
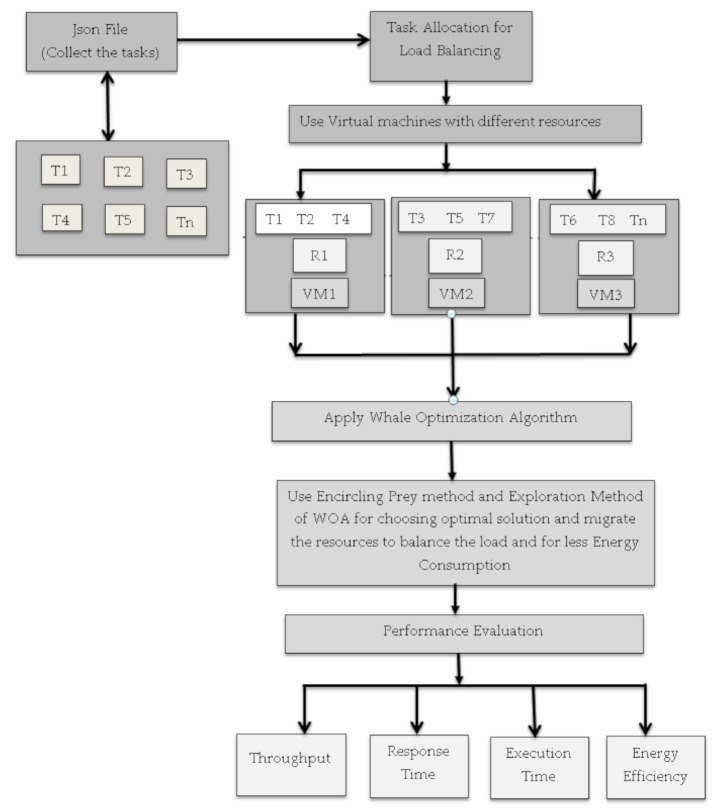
Workflow diagram of the proposed system.

The optimized energy efficient resource management model for cloud computing architectures uses certain algorithms for load balancing that help reduce the energy consumption effectively while balancing the loads among the servers [42]. The central aim of the system is to perform load balancing by the load transfer from overloaded servers to under loaded servers as well as perform task scheduling efficiently that helps in minimizing the energy consumption in a cloud environment. Although many such systems have been designed to achieve this purpose, the consumption of energy can be significantly decreased by applying a combination of algorithms. The proposed system utilizes the Cloud Analyst tool to model and imitate the cloud infrastructures. This work follows a certain procedure to perform balancing of workload and resource allocation to decrease the consumption of energy. Figure 3 depicts the workflow of the newly designed energy efficient load balancing system.

In the Figure 3 workflow, first of all there is the Json file which provides parameters to different tasks and collect their inputs in the form of the task then there is scheduler that allocates the task to different virtual machines based on the load given to that particular machine. After that a proposed algorithm checks the performance of different machines based on their functions and then redistributes the resources to improve the performance of the system. 

The proposed optimized energy efficient load balancing model works by following certain algorithm steps. These methodology steps are as follows:

- Input the required number of parameters to create the number of tasks.
- Host the number of servers on each virtual machine to allocate the different resources on it.
- Use the data center to launch all the virtual machines on it and to allocate the storage for them.
- Use the optimization methods to best schedule the resources and to balance the load from one server to another by migrating the resources and to compute the energy consumption.
- We need to compute the pre-migrations and post migrations time for migration and energy consumption and execution time for load balancing on cloud.
- To build the GUI for each optimization methods with proper executing button by using we can fire the event to execute the optimization methods one by one.
- By executing all the proposed methods, we can come to identify the best method for the research work at hand.
- Perform all the proposed work by using Java programming and the Cloud Analyst toolkit.

### 3.2. Energy Consumption over the Cloud System

Many models are available to calculate the energy consumed by servers, like CPU models, system models, and parallel system models [43]. Cloud systems use servers with many hardware parts, but an essential part of physical machines is the CPU, which consumes most of the energy. Software can be developed based on less CPU utilization, while other software can also manage the consumption. Based on this, we can find a solution to improve the energy consumption of a cloud system. 

Chedid et al. [43] have given an equation for the energy consumption of CMOS circuits:

(1) $$E\;\propto C_{eff\;}V^{2}f_{CLK}$$

where $C_{eff}$ is the effective switching capacitance of the operation, *V* is the supply voltage, and $f_{CLK}$ is the clock frequency and $f_{CLK}\propto \;\frac{{\left(V-V_{K\;}\right)}^{\alpha }}{V}$ is related to supply voltage Kumar [44].

In a cloud system, we assign loads to virtual machines which increase or decrease their CPU utilization and we can formulate that as follows:

(2) $$E\left(u\right)=\left(P_{max}-P_{min}\right)*\frac{u}{100}+P_{min}$$

Here $E\left(u\right)$ is the energy consumed by the CPU expressed as a utilization *u*% and *u* is the percent value of the processor utilization, where as $P_{max}$ and $P_{min}$ are the power consumption at maximum performance in Watt and at idle status, respectively.

Our proposed algorithm effectively schedules the task, so that server load is appropriately maintained. When the server picks up the load for executing the tasks, that state of the server is at maximum power consumption while if there is no load on the server, the server is consuming minimum power. We assumed that $P_{min}$, the minimum power consumption on an ideal server is 200 Watts and the maximum power consumption considered, $P_{max}$, is 400 Watts. It is better to maintain 70–80% CPU utilization otherwise, there will be a compromise in system performance. 

### 3.3. Mathematical Model

In a cloud system, there are basically two concepts: one is resource allocation and the second is task allocation. Resources allocation means allocation of virtual machines that act as resources of the cloud system. These virtual machines are allocated to tasks based on some assumptions like which machine is ideal, machines performing the same kind of task and more. The second is task allocation, where tasks are those assignments that come from consumers and we have to perform that on our virtual machines [44]. Every task comes with some parameters like its ID, arrival time, CPU utilization, etc. Resource allocation is done only if we have matching resources available for that particular task.

For minimizing the energy resource allocation of cloud system, a linear programming equation can be used as represented in Equation (3):

(3) $$Minimize\;E=\sum_{T=1}^{T}\sum_{i=1}^{m}E_{i}\left(T\right)$$

Here $E_{i}\left(T\right)=\left(P_{max}-P_{min}\right)*\frac{U_{i}\left(T\right)}{100}+P_{min}$ and:

$U_{i}\left(T\right)=\sum_{j=1}^{n}u_{\left(i,j\right)}$ ≤ peak load at time *T*, $\forall \;R_{i}\;\in R\;and\;\forall \;t_{j}\in T$
$u_{\left(i,j\right)}$ = 0; when task *j* is not assigned to node *R_i_*.
$u_{\left(i,j\right)}$ = $u_{\left(i,j\right)}$; when task *j* is assigned to node *R_i_*.

### 3.4. Algorithm Used for Load Balancing, Scheduling, Energy Efficiency

The proposed optimized energy efficient load balancing model is designed in various phases that are described in this section. Every phase employs certain algorithms to achieve load balancing as well as resource scheduling in the system. In the first phase PSO and CSA have been implemented. Then, after that in the second phase CSO and BAT was implemented for better resource utilization. Finally, the whale optimization algorithm has been implemented which gives the best results for optimizing the cloud system. This section discusses about these algorithms used in this system.

- PSO
- CSA
- CSO
- BAT
- WOA

These algorithms are discussed below:

*(i) Particle Swarm Optimization (PSO)*

**Figure 4:**
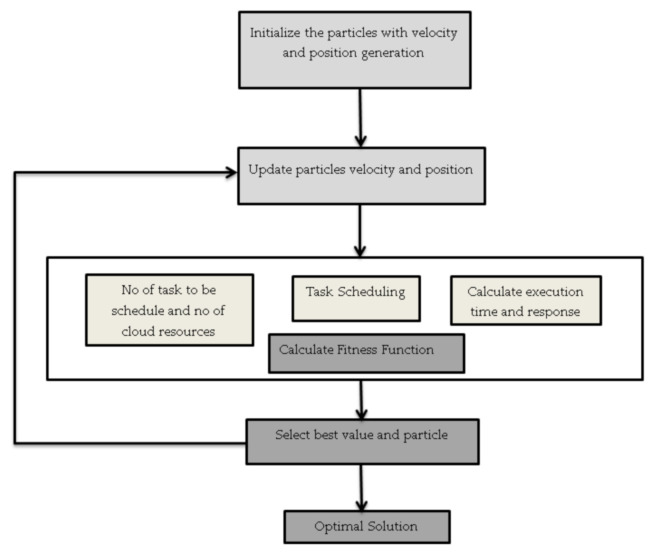
PSO algorithm.

Particle swarm optimization is a metaheuristic algorithm has inhabitants—called a swarm—of contender solutions known as particles [45]. These particles tend to progress around the search-space in accordance with the following statement: the particle movement is controlled by their own best known position and the entire swarm’s finest position. When the better locations are found, then they come to guide the swarm movements. Many empirical evidences have showed that this algorithm is an effective optimization tool. The workings of PSO are also described by various authors Bala [7], Satapathy et al. [45], and Liu et al. [46]. The above algorithm is explained in Figure 4 below.

Pseudo code
For each particle Initialize particle End **Do**  **For** each particle    Calculate Fitness value   If fitness value is better than the best fitness in history   Set current value as new pbest. Set current value as new pbest. **End**Choose the particle with best fitness value of all particles as the gbest  **For** each particle   Calculate particle velocity   Update particle position  **End****While** max iterations or minimum error criteria is not attained

*(ii) Cuckoo Search Algorithm (CSA)*

This is an optimization algorithm that is utilized to perform load balancing inspired by the required or compelled parasitic behavior of some cuckoo species that lay their eggs in the nests of some other host birds of some other species [10]. The nest which will be best with high quality of eggs carries out the next generation. The number of host nests is constant or fixed. The host nest he wants to throw out eggs to make a new nest solution [47,48].

Pseudo code
**Begin**     Objective function f(x)     Generate initial population of n host nest     Evaluate fitness and rank eggs    **While** (t > MaxGeneration) or Stop criterion     T = t + 1     Get a cuckoo randomly/generate new solution by Levy flights     Evaluate quality/fitness, F_i_     Choose a random nest j    **If** (F_i_ > F_j_)      Replace j by the new solution    **End if**      Worst nest is abandoned with probability P_a_ and new nest is built      Evaluate fitness and Rank the solution and fit current best   **End while**      Post process results and visualization**End**

*(iii) Cat Swarm Optimization (CSO)*

This algorithm is an intelligent algorithm that gets its motivation from the natural behavior of cats – their tracing mode and the seeking mode. In this algorithm, initially the number of cats that are to be used is decided, then the cats are applied in the algorithm to resolve the issues. To merge the two modes in CSO, a mixture ratio needs to be defined that must be a small value [11]. The action of cats normally comprises spending their most of the time resting but attentive, with a relaxed and intended approach called seeking mode. There is another mode called tracing mode when hunting targets as they travel with great velocity. This technique is inspired by the behavior of cats to get a difficult answer for optimal explanations. Complete mathematical models have been explained by various authors Bilgaiyan et al. [49], and Ahmed et al. [50].

Pseudo code
Randomly initialize cats. **While** (is terminal condition reached)   Distribute cats to seeking/tracing mode   **For** (i = 0; i < NumCat;i++)    Evaluate Fitness for cat.    **If** (Cat_i_ in seeking mode) THEN    Search by seeking mode process.    **Else**     Search by tracing mode process.    **End** **End For**  **End While**

*(iv) Bat Algorithm*

This is a metaheuristic algorithm inspired by the reverberation characteristic of bats with variation in pulse rates of emission as well as loudness Cai et al. [51]. It is created to perform global optimization tasks. Bats follow some rules [52,53]:

➢ Bats use echolocation to find the distance between food sources but they also have knowledge of the difference between background barriers and food.
➢ Bats search for their food with velocity v_i_ at position x_i_ with fixed frequency f_min_ with varying wavelength. They adjust their position and wavelength based on the target.

The pseudo code of the bat algorithm is as follows:

Pseudo code
Objective function f(x),x = [x_1_, x_2_, …, x_d_]^T^  Initialize the bat population x_i_(i = 1,2, … n) and v_i_  Define Pulse frequency f_i_ at x_i_ initialize pulse rates r_i_ and the loudness A_i_ **While** (t< Max numbers of iterations)     Generate new solutions by adjusting frequency     And updating velocities and locations/solutions [(1)]  **If** (rand>r_i_)      Select a solution among the best solutions      Generate a local solution around the selected best solution  **End if**
      Generate a new solution by flying randomly  **If** (rand < A_i_ and f(x_i_) < f(x*))      Accept the new solutions      Increase ri and reduce Ai   **End if**    Rank the bats and find the current best x***End while**    Postprocess results and visualization

*(v) Whale Optimization Algorithm (WOA)*

**Figure 5:**
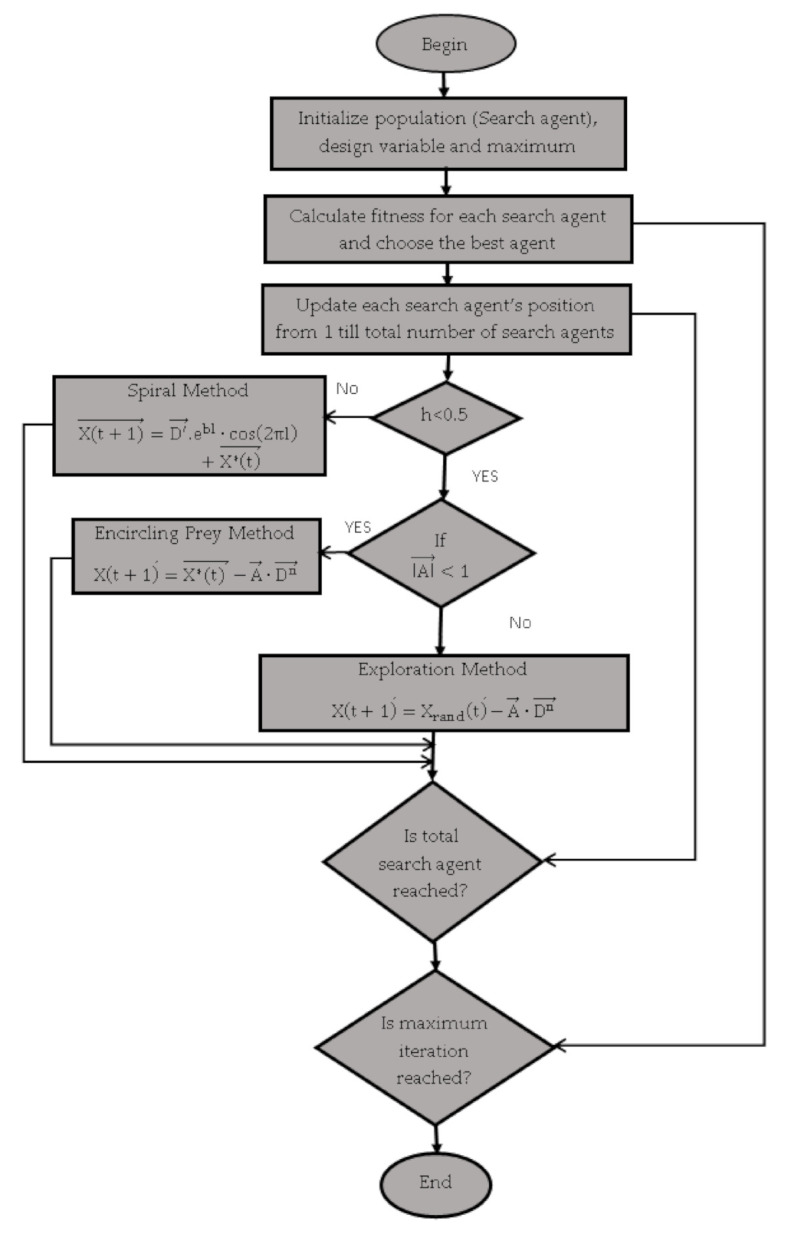
Flowchart of the whale optimization algorithm.

Whales can find out the location of their prey and then encompass them. Since the situation of the ideal plan in the pursuit space isn’t known earlier, the WOA algorithm considers that the present current best up-and-comer arrangement is the objective prey or is near the ideal solution [13]. When a best representative is defined then other one will also try to change their values according to the best one. The mathematical model has been explained by Thennarasu et al. [54]. This algorithm is explained in Figure 5 below.

The pseudo code of Whale Optimization algorithm (WOA) is as follows:

Pseudo code
Randomly initialize the whale populationEvaluate the fitness values of whales and find out the best search agent X^*^**While** t < t_max_  Calculate fitness function for each agent  **For** each search agent  **If** h < 0.5 where h is the random number between 0 and 1 then    **If** $\vec{\left|A\right|}<1$  then $\vec{X\left(t+1\right)}=\vec{X^{*}\left(t\right)\;}-\vec{A}\cdot \vec{D^{n}}$ ……………………………………**Equation (1)**    **Else  if **$\left|A\right|\geq 1\;then\;\vec{X\left(t+1\right)}=\vec{X_{rand}\left(t\right)}-\vec{A}\cdot \vec{D^{n}}$ ……………………………………**Equation (2)**     **end If**  **Else If** h ≥ 0.5 then    $\vec{X\left(t+1\right)}=\vec{D^{\prime }}.e^{bl}\cdot cos\left(2\pi l\right)+\vec{X^{*}\left(t\right)}$ ………………………………………………**Equation (3)**  **End If**
  **End For**Evaluate the fitness of X(t + 1) and updates X′**End While**

In WOA, h is the random number between 0 and 1. In Equation (1), $X\left(t+1\right)$ is the updated position, X* (t) is the best solution and method A = (2*a*r)-a where a is linearly decreased from 2 to 0 depends on max iteration number shrinking encircling and r is random vector between [0,1] and D = $\left|\left(2*r*X^{*}\left(t\right)\right)-\left(X\left(t\right)\right)\right|$ where X (t) is position vector

In Equation (2), D = $\left|\left(2*r*X_{rand}\left(t\right)\right)-\left(X\left(t\right)\right)\right|$ and $X_{rand}\left(t\right)$ is position vector. In Equation (3), b defines the logarithmic spiral shape, L is random number between −1 and 1 and D = $X^{*}\left(t\right)-X\left(t\right)$.

### 3.5. Simulation Toolkit

Cloud Analyst is a simulation toolkit which provides modelling as well as simulation of the core functionality of the cloud such as event processing, creation of cloud entities, communication between varying entities, etc. This toolkit provides many services and features:

➢ Test the services of applications in a reoccurring and controlled environment.
➢ Performs experiments with varying workload mix and with different performance scenarios on imitated infrastructure for development.
➢ Tune the system limitations before deployment of apps in an actual cloud.
➢ It is a complete platform for modelling cloud’s service agents, provisioning, and, allocation strategy.
➢ It provides imitation of a cloud-environment which can inter-connect resources of both public as well as private domains.
➢ Virtualization engine availability that helps in creation as well as management of several independent virtual services on a DC node.

### 3.6. Parameters Used in Toolkit

**Figure 6:**
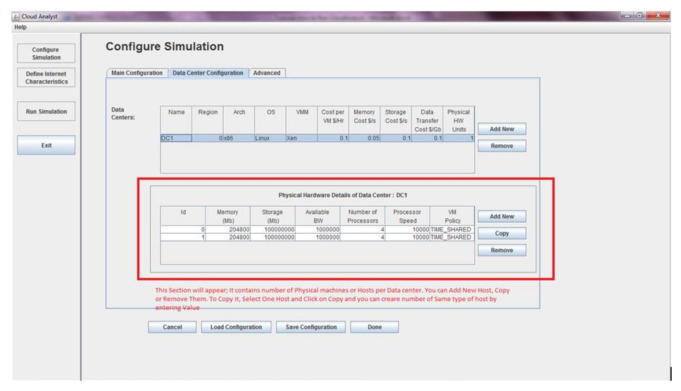
Parameter Setting in Cloud Analyst.

There is datacenter tab in the Cloud Analyst simulation toolkit which allows defining the configuration of the datacenter. There is need to give values to various parameter fields like name, region, operating system, number of servers, etc. Under data center, a second table appears which require more parameter values like machine id, memory, storage, processor speed, VM allocation policy as shown Figure 6. Other than this there are some important parameters that are:

➢ *User Grouping Factor*: This parameter is used to guide simulator that in a single bundle of traffic, how many users will be treated simultaneously.
➢ *Executable instruction length (in bytes)*: Simulator execute instructions based on length setting under this parameter.
➢ *Load balancing policy*: This parameter is used to allocate request to various virtual machine based on policy setting.
➢ *Request Grouping Factor*: Simulator treat multiple requests for single unit of processing using this parameter.

**Figure 7:**
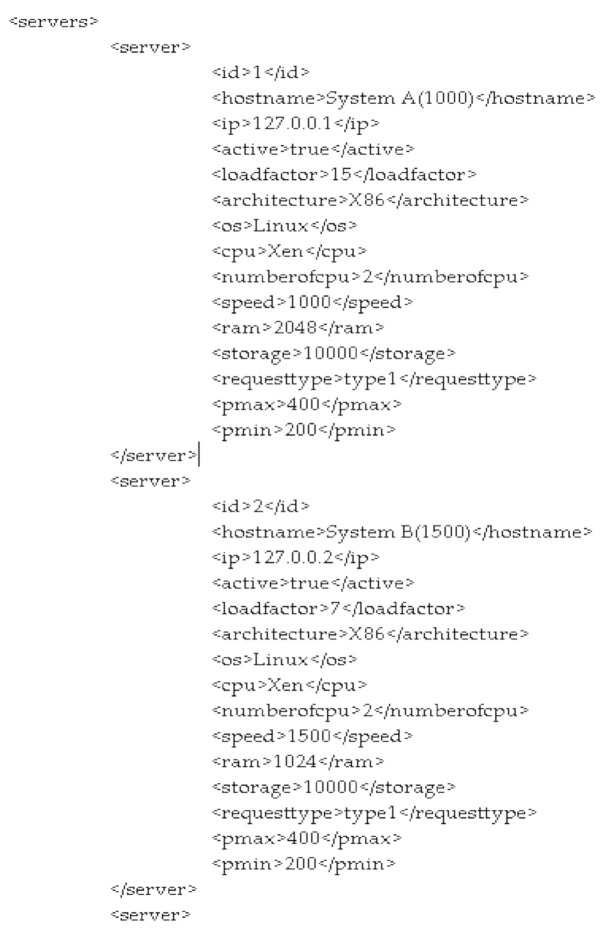
Servers specification in XML File.

The data center contains various physical servers which all have different kinds of properties of resources like storage, CPU, speed, storage, architecture, etc. as shown in the XML configuration file (see Figure 7). In the proposed algorithm, we have considered eight servers with different configurations, meaning that some servers have 2 GB RAM, some have 3 GB RAM. Similarly, few servers are of 32-bit architecture, others are of 64-bit architecture. These values are used when the system load the machines. For the proposed WOA algorithm, the XML file also includes Pmin and Pmax values for each server because our algorithm is not only scheduling tasks but also calculating energy consumption by that particular machine during the task allocation as per Equation (4):

Power Consumed = Pmin + (Pmax − Pmin) * Utilization
(4)

where Pmin is set as minimum power consumed by the server in its ideal state and Pmax is the maximum power consumed by the server in its utilization state [55] and these values are set in the configuration file. These values have been set as 200 and 400 as min and max values, respectively, because it has found that most servers consume near to 200 Watts of power when they are in an ideal state and if they contain a large number of resources then they can consume nearly 400 Watts of power [56,57,58].

Based on all these values, when our proposed WOA algorithm schedules the tasks to different virtual machines, the corresponding energy consumption is calculated based on the utilization of resources of that server [59].

### 3.7. Simulation Output Screen

**Figure 8:**
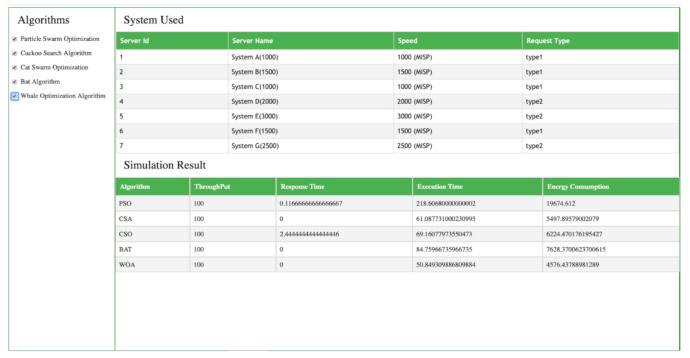
Algorithm results with seven servers.

**Figure 9:**
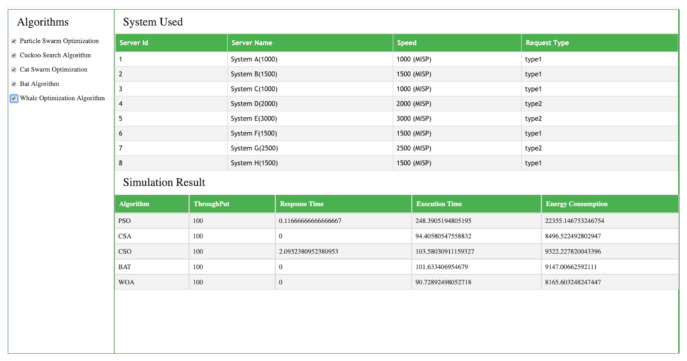
Algorithm results with eight servers.

The following snapshots show the result in terms of response time, execution time, throughput, and, energy consumption when PSO, CSO, BA, and, CSA, WOA are applied to multiple numbers of servers. Figure 8 and Figure 9 shows the simulation results of PSO, CSO, BA, and, CSA, WOA algorithms using seven and eight servers, respectively.

## 4. Results and Discussion

In this section, a comparison of different algorithms with our proposed algorithm is presented.

### 4.1. Seven Servers

Figure 8 displays the simulation result of the PSO, CSA, CSO, BAT and WOA algorithms when we are use seven servers of different capacity. Each server has different RAM, imps speed and different numbers of virtual machines. Cloud Analyst simulator shows results of these algorithms for various parameters like throughput, response time, execution time and energy efficiency. The simulation results showed that PSO and CSO have 0.116 and 2.466 ms response times but the CSA, BAT and WOA algorithms take 0 ms. There is big variation in energy consumption between the various algorithms and WOA gives the best result. When we implement PSO, the cloud system consumes 19,624,612 Joules, in the case of CSA the energy consumption is 5492 Joules, CSO consumes approximate 6224 Joules, BAT consumes 7828 Joules but when WOA is implemented, the energy consumption is reduced to 4536 Joules. Similarly, in the case of the execution time parameter, PSO takes 218 ms, CSA takes 60 ms, CSO takes 69 ms, BAT takes 84 ms, but WOA takes only 50 ms for task execution. This finding shows that WOA outperforms PSO, CSA, CSO, BAT in terms of throughput, response time, execution time and energy efficiency.

### 4.2. Eight Servers

Figure 9 represents the simulation results of PSO, CSA, CSO, BAT and WOA regarding throughput, response time, execution time and energy consumption using eight different kind of servers. The simulation results show that PSO and CSO have 0.116 and 2.095 ms response times, but the CSA, BAT and WOA algorithms take 0 ms. The time taken by the different algorithms for task execution is also good in the WOA algorithm as compared to the others. WOA takes only 90.72 ms, while PSO takes 248.390 ms, CSO uses 94.405 ms, CSA needs 103.633 ms, and Bat takes 101.633 ms. When we compare energy consumption using the WOA algorithm as compared to the other algorithms is also gives the best results. Energy consumption in the case of PSO is 22,355.16 Joules while for CSO it is 8496.522 Joules, for CSA it is 9322.227 Joules, BAT consumes 9147.006 Joules and WOA consumes 8165.603 Joules which is much less as compared to PSO, CSA, CSA and BAT. The above results demonstrate that WOA beats PSO, CSA, CSO, BAT in terms of throughput, response time, execution time and energy consumption.

### 4.3. Load Balancing

Table 2 shows the comparison of response time, execution time and energy consumption of PSO, CSO, CSA, BAT and WOA that has been implemented in various phases of research. The load balancing, throughput and response time of each algorithm has been tested, and the results show that the throughput is same—100 ms—for all the algorithms, but the response time of PSO is 0.116, of CSO it is 0 ms, for CSA it is 2.095 ms, for BAT it is 0 ms and for WOA it is also 0 ms. The execution time taken by machines for PSO is 248.390 ms, for CSO it is 94.405 ms, for CSA it is 103.633 ms, for Bat it is 101.633 ms and for WOA it is 90.7289 ms. The energy consumption of PSO is 22,355.16 Joules, for it CSO is 8496.522 Joules, for CSA it is 9322.227 Joules, BAT consumes 9147.006 Joules and WAO consumes 8165.603 Joules. It has thus been found that overall the whale optimization algorithm gives the best results as compared to the PSO, CSA, CSO and Bat algorithms for load balancing, resource scheduling and energy optimization. Hereafter, in terms of response time, execution time, energy consumption, WOA showed better performance as compared to the rest of algorithms used in the study.

### 4.4. Energy Consumption

**Figure 10:**
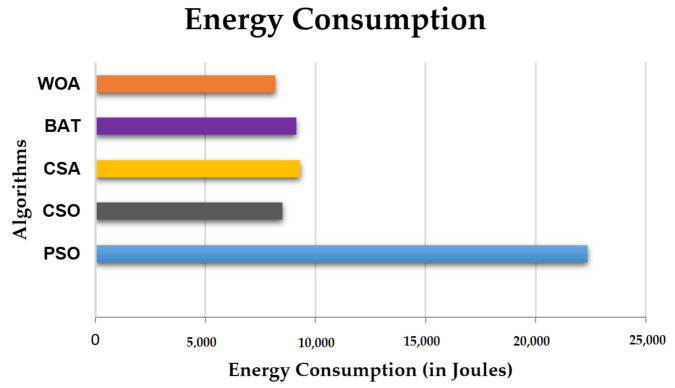
Comparison of the energy consumption of the PSO, CSO, CSA, BAT with WOA algorithms.

Figure 10 depicts the graphical comparison of energy consumed by the PSO, CSO, CSA, BAT and WOA algorithms when large numbers of servers are active. It shows that when we use the WOA algorithm, the energy consumption is 8165.603 Joules, while in CSO implementation, the energy consumption is 8496.522 J, so WOA gives a 4% better result as compared to CSO. When we use the BAT algorithm, the cloud system consumes 9147.006 Joules of energy, oo WOA is 12% better than BAT. CSA implementation requires 9322.227 Joules of energy consumption, so WOA is 13.22 % better. WOA is 92 % better as compared to PSO because PSO consumes 22355.146 Joules of energy. Therefore, we can say WOA outclasses all the other algorithms in terms of energy consumption.

### 4.5. Execution Time

**Figure 11:**
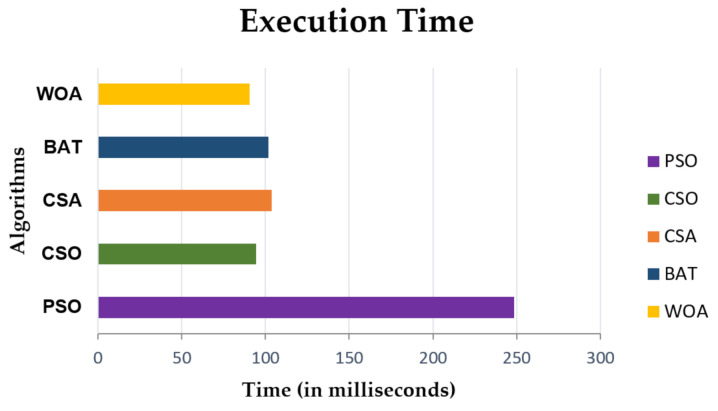
Comparison of execution time of PSO, CSO, CSA, BAT with WOA.

Figure 11 shows the execution times of the PSO, CSO, CSA, BAT and WOA algorithms using a graphical comparison. The results show that WOA takes 90.7289 ms, while CSO takes 94.405 ms which shows that WOA gives 4% better execution time results compared to CSO. 

Similarly, when we compare WOA with the BAT algorithm, WOA is 11% better because BAT takes 101.633 ms for task execution. WOA is also compared with CSA and PSO. WOA is 13% better than CSA, and 92% better than PSO. CSA takes 103.580 ms execution time and PSO takes 248.390 ms for task execution. From this we can conclude that WOA needs less execution time as compared to the rest of the algorithms.

For the testing environment, different servers with different speeds, RAM and bandwidth are considered with different numbers of jobs. As the results show, the WOA algorithm gives the best results as compared to the other algorithms. In the implementation of WOA, energy consumption used by cloud systems is much less and response time and execution time is also less as compared to other algorithms. With less energy consumption and less response and execution time WOA is cost effective in comparison with other algorithms.

## 5. Conclusions and Future Work

Cloud computing has appeared to be one of the most sought after network configurations to which IT and other data storage facilities are leaning with a cost effective approach. The utilization of cloud computing has increased significantly which indeed has led to traffic clogging at the web servers. Due to the large number of clients, the demand for resources has also increased thereby creating an imbalance in the distribution and allocation of resources. The proposed work addresses the problem of load balancing and resource scheduling with a cost effective approach. It is also seen that implementation of different algorithms for effective resource allocation and balancing of workload in cloud causes great a consumption of energy. To harness greater output, firstly we have implement algorithms like PSO, CSO, CSA and BAT and check their result using Cloud Analyst simulator. Finally, our proposed work uses the whale optimization algorithm which lowers the energy consumption rate and also decreases the execution time as compared with other algorithms.

One of the limitations of the present work is that we have not compared WOA using other parameters like cost, makespan, etc. In future work, we will compare different algorithms with different parameter settings. We can implement various meta-heuristics techniques for the improvement of the work. Secondly, we can also move towards different computing system such as fog computing along with cloud for load balancing and energy efficiency. We can also implement our proposed algorithm in a real cloud system like Amazon’s EC2, Microsoft’s Azure etc. rather than just use the Cloud Analyst simulator.

## Figures and Tables

**Table 1:** Comparison of Various Techniques.

Author	Technique	Benefits	Limitation
Adhikari et al. [35]	LB-RC	Achieve less execution cost with QoS parameter	Qualities of task deployment policies is not considered
Jena et al. [36]	QMPSO	balances the load by reassigning the load to the appropriate VMs by considering the fitness value of each VMs	It is limited to independent tasks
Golchi et al. [37]	hybrid firefly and IPSO	Response time of task has been increased	Energy efficiency not maintained
Haidri et al. [38]	CPDALB	Provide better result of load balancing in heterogeneous environment	Various other parameter also need to be focussed other than Load balancing
Pourghaffari et al. [33]	EDF-VD	Give better results of load balancing by spiting task	Much more better results can come for task scheduling
Kaur et al. [39]	TSFPA	Makespan of tasks has been reduced as compared to other techniques	Load balancing between tasks need to be consider
Mishra et al. [40]	ACO-Fuzzy	Optimize cost and provide optimal computer network path for resource allocation	Multiobjective optimization of resources, energy consumption and task migration need to be implement.
Xiaolong et al. [41]	MTSS	Give the result of better network utilization, reduction in packet loss	Not consider various load cnditions

**Table 2:** Comparison of the proposed algorithm with the other implemented algorithms.

Algorithms	Parameters		
	Response Time	Execution Time	Energy Consumption
PSO	0.116	248.390	22,355.146
CSO	0	94.405	8496.522
CSA	2.095	103.580	9322.227
BAT	0	101.633	9147.006
WOA	0	90.7289	8165.603

## Data Availability

The data that support the findings of this study are available from the corresponding author, upon reasonable request.
